# Comprehensive Toxicological Evaluation of 3D-Printed Hydroxyapatite (3DPHA) for Bone Grafting Applications

**DOI:** 10.3390/ijms27020636

**Published:** 2026-01-08

**Authors:** Faungchat Thammarakcharoen, Autcharaporn Srion, Waraporn Suvannapruk, Watchara Chokevivat, Wiroj Limtrakarn, Jintamai Suwanprateeb

**Affiliations:** 1 Biofunctional Materials and Devices Research Group, National Metal and Materials Technology Center (MTEC), National Science and Technology Development Agency (NSTDA), Khlong Nueng, Khlong Luang, Pathum Thani 12120, Thailand; faungcht@mtec.or.th (F.T.); autchars@mtec.or.th (A.S.); waraporn@mtec.or.th (W.S.); watcharc@mtec.or.th (W.C.); 2 Department of Mechanical Engineering, Faculty of Engineering, Thammasat University, Khlong Nueng, Khlong Luang, Pathum Thani 12120, Thailand; limwiroj@engr.tu.ac.th; 3 Thammasat University Center of Excellence in Computational Mechanics and Medical Engineering, Thammasat University, Khlong Nueng, Khlong Luang, Pathum Thani 12120, Thailand

**Keywords:** toxicology, 3D-printed hydroxyapatite, bone graft, binder-jet 3D printing, biocompatibility

## Abstract

Binder jet 3D printing combined with a low-temperature phase transformation process has emerged as a promising route for producing 3D-printed hydroxyapatite (3DPHA) scaffolds with controlled architecture for bone grafting applications. However, the toxicological profile of this unique binder jet-derived material has not yet been established. In this study, we conducted a comprehensive compositional and toxicological assessment of 3DPHA fabricated via the calcium sulfate transformation route. The material exhibited phase-pure hydroxyapatite (HA) with a Ca/P ratio consistent with non-stoichiometric HA and low levels of trace elemental impurities. In vitro assays revealed no cytotoxic, irritant, sensitizing, or mutagenic effects. This work provides a standardized toxicological and compositional safety validation of 3DPHA. By linking compositional purity to biological safety and demonstrating compliance with international benchmarks, this study establishes a regulatory foundation confirming that 3DPHA is chemically pure, biologically safe, and ready for clinical translation as a bone-graft material.

## 1. Introduction

The management of critical-size bone defects resulting from trauma, infection, or tumor resection necessitates the development of bone-substitute materials, given the known limitations and disadvantages associated with autografts and allografts. Calcium phosphate (CaP) ceramics represent the cornerstone of synthetic bone-graft substitutes due to their intrinsic osteoconductive properties and chemical resemblance to the mineral phase of human bone and hard tissues [1,2,3]. Among the various CaP phases, HA is regarded as the most widely used bioceramic material for bone-grafting and bone-tissue engineering applications, as it closely mimics the composition of natural bone mineral [4,5].

For HA to function optimally as a bone graft or bone scaffold, its architecture must permit rapid integration with surrounding host tissue. This requires porous structures that facilitate cell attachment, proliferation, nutrient exchange, and vascularization. Studies have shown that porous HA is more osteoconductive and resorbable than dense HA, as the increased surface area enhances cellular interactions [6,7]. Furthermore, strong mechanical interlocking with regenerating bone is achieved when pores provide sufficient interconnectivity and appropriate geometry [8,9]. Achieving effective bone graft or scaffolds, therefore, requires precise control over architectural parameters such as pore size, interconnectivity, and permeability, which are difficult to achieve using conventional ceramic processing methods.

Additive manufacturing (AM), or three-dimensional (3D) printing, overcomes traditional fabrication limitations by enabling layer-by-layer construction of bioceramic grafts with precisely controlled geometries [10,11,12,13]. This technology also allows for the production of complex, patient-specific bone grafts and scaffolds that match the morphology of defects. Several AM processes have been employed for biomedical applications, including powder bed fusion (PBF), material extrusion (MEX), vat photopolymerization (VPP), and binder jetting (BJT). The BJT technique is particularly suitable for processing bioceramic materials [14,15,16,17,18]. Although BJT typically yields parts with lower mechanical strength compared to other 3D printing methods, mainly due to high porosity and weak interparticle bonding, its major advantage lies in its high speed and the ability to fabricate complex structures at low temperatures via layer-by-layer deposition of a liquid binder onto a powder bed, without the need for polymer carriers or high-temperature post-processing like slurry-based methods such as stereolithography (SLA) or digital light processing (DLP), and melt-based techniques like fused deposition modeling (FDM) that could potentially compromise bioactivity [19,20,21,22].

Previously, BJT using calcium sulfate-based powders, followed by a low-temperature phase transformation process, has been developed by our research group to fabricate 3DPHA scaffolds for bone-grafting applications [19,20,21,22]. This proprietary route enables the formation of HA from printed calcium sulfate through a dissolution–reprecipitation mechanism, producing constructs with previously demonstrated low crystallinity, osteoconductivity, and controlled resorbability. Previous studies from our group have reported the fabrication process, mechanical behavior, and in vitro osteogenic potential of 3DPHA [23,24,25,26]. Furthermore, 3DPHA has already been evaluated in pilot clinical studies for alveolar ridge preservation and bone regeneration, demonstrating promising outcomes and safety [27,28]. However, despite these encouraging outcomes, no systematic toxicological or chemical safety evaluation of 3DPHA has been published to date. Given the distinct low-temperature processing route, residual binder content, and potential for chemical impurities, a comprehensive toxicological assessment is necessary to confirm its safety and regulatory suitability.

Therefore, this study provides an integrated toxicological and compositional evaluation of 3DPHA fabricated via the calcium sulfate phase transformation route developed by our group. Specifically, this work aims to assess the physicochemical composition and preliminary biological safety of binder-jetted calcium sulfate constructs after low-temperature transformation into HA. The physicochemical characterization included analyses of phase composition, phase purity, Ca/P molar ratio, and trace heavy metal content to verify the structural and chemical stability of the transformed material. In addition, selected in vitro biological tests, including assays for cytotoxicity, cell proliferation, osteogenic differentiation, irritation, sensitization, and genotoxicity, were conducted to provide initial insight into the safety and biocompatibility of 3DPHA. Collectively, these evaluations determine whether 3DPHA exhibits acceptable compositional stability and biological safety, supporting its continued development for bone-grafting applications. Despite prior evidence of the clinical feasibility of 3DPHA, the toxicological safety of 3DPHA remains an unresolved problem in the field. No published work has yet established whether this manufacturing route produces a material that meets international chemical-safety and biocompatibility standards. Accordingly, this study was designed to answer three specific research questions: (1) Does 3DPHA exhibit chemical purity, Ca/P stoichiometry, and trace-element levels within biomedical safety limits? (2) Does it demonstrate the absence of cytotoxic, irritant, sensitizing, or genotoxic effects in accordance with ISO 10993 and OECD guidelines? (3) Can these findings confirm that 3DPHA is toxicologically safe and compositionally stable for regulatory approval and clinical translation? Addressing these questions clarifies the material’s safety profile and establishes a standardized toxicological foundation for its future clinical use.

## 2. Results

**Figure 1:**
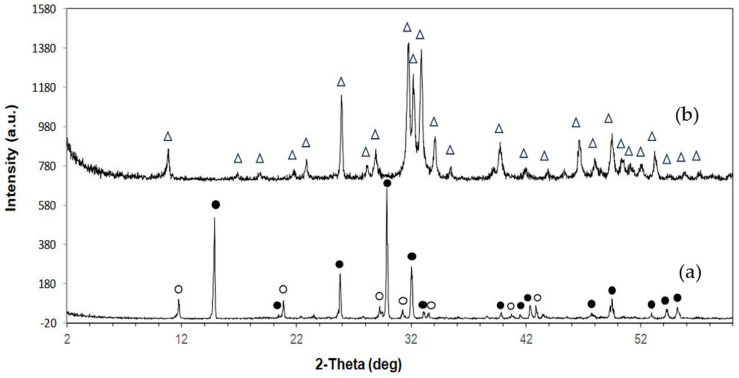
XRD pattern of 3D-printed samples: (**a**) As-printed; (**b**) 3DPHA. Symbols: • Bassanite; O Gypsum; Δ Hydroxyapatite.

**Figure 2:**
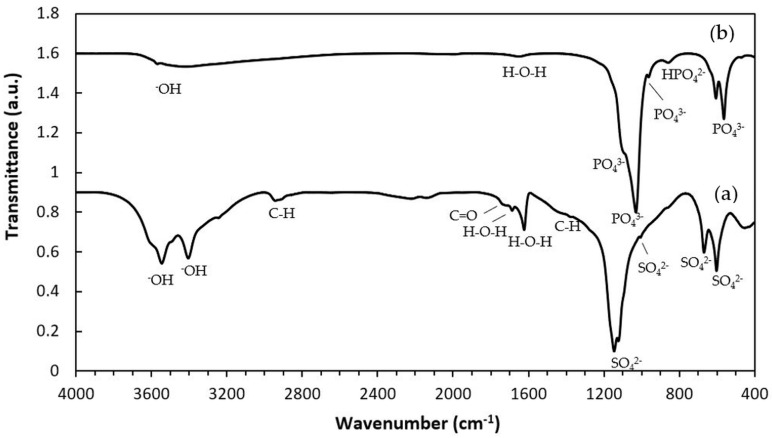
FTIR spectra of 3D-printed samples: (**a**) As-printed; (**b**) 3DPHA.

The XRD patterns of the samples are shown in Figure 1. The as-printed specimen displayed diffraction peaks characteristic of bassanite (calcium sulfate hemihydrate), along with minor peaks corresponding to gypsum (calcium sulfate dihydrate). After undergoing the phase transformation process, only peaks attributed to HA were observed, confirming the complete conversion from calcium sulfate to HA. Additionally, the XRD peaks appeared generally broad, indicating the low crystallinity of the material. The quantitative phase compositions before and after transformation are summarized in Table 1. Figure 2 shows a representative FTIR spectrum of the as-printed sample and 3DPHA after transformation. The FTIR spectra of the as-printed sample show the characteristic spectral bands of both bassanite and gypsum [28,29], comprising H_2_O stretching (νH_2_O) for bassanite near 3495 and 3615 cm^−1^ and for gypsum at 3245, 3405, 3495, and 3545 cm^−1^, secondary vibrational modes attributed to interactions involving water bending and sulfate vibrations of bassanite at 2142 cm^−1^ and 2219 cm^−1^, H_2_O bending (δH_2_O) vibrations at 1622 cm^−1^ for bassanite and at 1622 and 1686 cm^−1^ for gypsum, SO_4_^2–^ ν4 asymmetric bending vibration at 667 cm^−1^ and 601 cm^−1^, SO_4_^2–^ ν2 symmetric bending vibration at 451 cm^−1^, SO_4_^2–^ ν3 asymmetric stretching vibration at 1005, 1121–1144 cm^−1^, SO_4_^2–^ ν2 asymmetric bending vibration at 859 cm^−1^. Some minor bands not assigned to calcium sulfate were observed, with absorption peaks around 2942, 1738, and 1378 cm^−1^, which may be attributed to organic compounds, indicated by C–H stretching and bending vibrations as well as C=O stretching. These bands are distinct from typical calcium-sulfate peaks, which are primarily related to sulfate group vibrations and water of hydration. After transformation, the 3DPHA displayed main spectra bands of HA at 1029–1126 cm^−1^ corresponding to the ν3 mode of PO_4_^3–^, НРO_4_^2–^ bands at 858 cm^−1^, ν4 mode of PO_4_^3−^ at 604, 562, 470 cm^−1^, ν1 mode of PO_4_^3–^ band at 962 cm^−1^, OH- bands at 3700–2400 cm^−1^ and ν2 mode of Н_2_О band at 1658 cm^−1^ [30].

Elemental analysis conducted using ICP-OES revealed that the Ca/P molar ratio of the 3DPHA was 1.28, while those of commercial allografts and xenograft were about 1.50 (Table 2). Assessment of potentially toxic heavy metals indicated that the 3DPHA contained cadmium (Cd), lead (Pb), arsenic (As), and mercury (Hg) at concentrations of 0.05, 0.42, 0.11, and <0.020 mg/kg, respectively. The main heavy metals found in commercial bone grafts are Pb, ranging from approximately 0.85 to 2.38 mg/kg, and As between 0.03 and 0.24 mg/kg. Meanwhile, Cd and Hg levels are limited to below 0.020 mg/kg.

**Figure 3:**
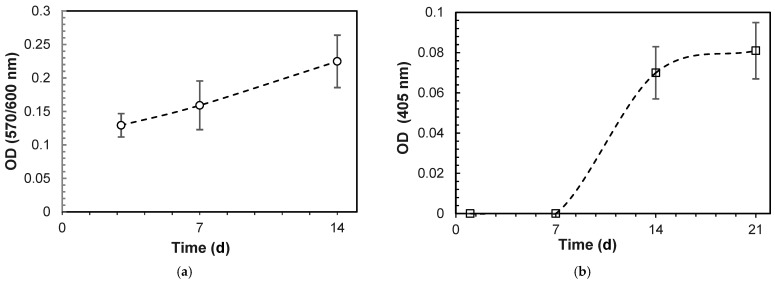
Cell proliferation and differentiation on 3DPHA by: (**a**) alamar blue assay; (**b**) ALP activity (Mean ± SD, n = 6).

The initial cytotoxicity assessment using the MTT assay on L929 fibroblasts indicated that the 3DPHA extracts exhibited no cytotoxic potential, with cell viability consistently measured at above 70% (Table 3). The bioactivity was evaluated using MC3T3-E1 pre-osteoblast cells. Cell proliferation, quantified via the Alamar Blue assay, demonstrated a time-dependent increase in metabolic activity (optical density, OD) on the 3DPHA surfaces across the various culturing periods (Figure 3a). Furthermore, the osteogenic potential was confirmed by an increase in ALP activity over time, beginning from Day 7 onward (Figure 3b). In the in vitro irritation test, the tissue viability of the 3DPHA and the negative control exceeded the 50% threshold, indicating a non-irritant response, contrasting with the irritant response observed for the positive control (Table 4). In the in vitro sensitization test, the dose-finding assay for the 3DPHA extract and negative control failed to reduce cell viability at any tested concentration, precluding the determination of the CV75 value (Table 5). Conversely, the positive control (DNCB) showed a concentration-dependent reduction in viability, allowing for a CV75 determination of 3.55 μg/mL. Subsequently, expression levels of key activation markers (CD86 and CD54) were assessed using the highest extract concentration for 3DPHA (50% (*v*/*v*). The 3DPHA extracts showed consistent negative responses for both CD86 and CD54 expression (Table 6), indicating an absence of potential to induce skin sensitization following a 24 h exposure. Finally, the genotoxicity assessment via the Ames test demonstrated no mutagenic effects in the 3DPHA extracts across all five standard *Salmonella typhimurium* strains (TA1535, TA1538, TA98, TA100, and TA102). This result confirms that the 3DPHA material does not induce genetic mutations in these standard bacterial models (Table 7).

## 3. Discussion

The chemical characterization results for the 3DPHA provide crucial insights into its material characteristics and initial safety profile, particularly when assessed against the established criteria for bone graft substitutes like ASTM F1185 Standard Specification for Composition of Hydroxylapatite for Surgical Implants [31] or ISO 13175 Implants for surgery-Calcium phosphates [32] or regulatory requirements like MDR and USFDA. Overall, the phase purity of HA should be ≥95% and the heavy metal content is referenced to USP <232> for limits.

In this study, XRD analysis of the as-printed sample revealed that bassanite (calcium sulfate hemihydrate) was the main phase, accompanied by a minor amount of gypsum (calcium sulfate dihydrate). The corresponding FTIR spectrum further confirmed these findings, showing characteristic sulfate vibrational bands (ν_3_ SO_4_^2−^ at 1120–1140 cm^−1^ and ν_4_ SO_4_^2−^ at 858–862 cm^−1^) together with broad OH^−^ stretching bands, consistent with the presence of both bassanite and gypsum. Collectively, the XRD and FTIR results confirm that the as-printed scaffolds consisted primarily of bassanite with a small fraction of gypsum. FTIR spectra of the as-printed calcium-sulfate samples also displayed absorption bands or organic matter at 2942, 1738, and 1378 cm^−1^, attributed to C–H stretching, C=O stretching, and CH_3_ bending vibrations, respectively, in addition to the inorganic sulfate bands. These bands correspond to the polyvinyl alcohol (PVA). During binder jetting, calcium-sulfate-based powders are consolidated primarily through a hydration reaction with the aqueous binder [27]. However, the kinetics of this reaction are typically too slow for the printing process itself, so both the binder and powder formulations usually include binders that provide initial strength to the “green” printed structure. Although the exact composition of the commercial aqueous binder used in the binder jetting machine is proprietary, it is known to consist mainly of nonhazardous components such as water, water-soluble polymers, a humectant such as 2-pyrrolidinone, and additives [27,33]. PVA is commonly used in binder jetting processes for consolidating powders due to their excellent printability, film-forming ability, and biocompatibility [25,26,27]. In addition to the binder, small amounts of PVA or similar water-soluble polymers are also incorporated into the commercial calcium-sulfate-based powders to function as an adhesive binder during consolidation [27]. Although organic components were not detected in the XRD pattern due to their amorphous nature, their FTIR signals confirm the presence of organic constituents in the as-printed state.

The presence of such an organic component can influence the subsequent low-temperature phase transformation to calcium phosphate. Specifically, it may retard ion exchange, modulate local pH conditions, and affect transformation kinetics, potentially influencing the resulting phase purity, crystal morphology, and porosity of the converted material. From a biological standpoint, any residual organic component, albeit non-hazardous to human use as stated by the manufacturer, could transiently affect cell adhesion and protein adsorption; although PVA is water-soluble, biocompatible, and widely used in biomedical formulations [34,35,36,37]. It has been previously demonstrated that binder-jetted calcium sulfate samples exhibited in vitro cytotoxicity toward MG63 (human osteoblast-like osteosarcoma) cells when untreated or heat-treated at low temperatures, such as 300 °C. However, samples heat-treated at higher temperatures between 500 °C and 1000 °C showed no cytotoxic effects, as this treatment resulted in the complete combustion of organic components [27]. Successful removal of cytotoxic organic residue using high-temperature treatment was also demonstrated in HA constructs made by other 3D printing techniques, including stereolithography (SLA) [21]. Despite this, since all organic constituents in both the binder and powder formulations are water-soluble, it is expected that these components dissolve during the transformation and cleaning processes, leaving only negligible residual amounts in the final 3DPHA structure. This dissolution minimizes potential cytotoxic risks associated with residual organics in the final hydroxyapatite scaffold.

Following the phase transformation, the XRD patterns of the 3DPHA samples displayed only characteristic peaks of HA, confirming the complete conversion of calcium sulfate to HA. Quantitative analysis revealed 100% phase purity, indicating a successful transformation. The broad XRD peaks of 3DPHA suggest a low crystallinity, which is similar to those of bone and bone-derived allograft or xenograft [38]. Supporting these findings, FTIR spectra of the transformed samples exhibited solely the expected phosphate (PO_4_^3−^) and structural hydroxyl (OH^−^) vibrational bands of HA, with no detectable signals from residual calcium sulfate or organic materials. Together, these XRD and FTIR results demonstrate that the binder jetting process, combined with controlled low-temperature transformation, effectively produced phase-pure HA with negligible organic residues. The material meets and exceeds the commonly required minimum of 95% HA content for surgical implant applications. The presence of a 5% calcium oxide (CaO) impurity phase was reported to significantly reduce the osteoconductivity of HA compared to phase-pure HA, confirming that the control over the phase purity of HA-based bioceramics is essential for optimal biological performance [39].

The elemental analysis conducted via ICP-OES yielded a Ca/P molar ratio of 1.28 for 3DPHA. This ratio deviates from the theoretical stoichiometry of 1.67 for HA indicating that 3DPHA is chemically a calcium-deficient hydroxyapatite (CDHA), where the structural integrity of HA is maintained, but the lattice incorporates calcium vacancies and/or substitution of OH^−^ with H_2_O or HPO_4_^2−^ to maintain charge neutrality due to a low crystallinity nature or a low crystallite size of 3DPHA, which was noted from broadened or less sharp peaks in the XRD pattern. This is confirmed by the FTIR band at 857 cm^−1^ that is the characteristic band of HPO_4_^2−^, indicating the presence of hydrogen phosphate ions substituting for PO_4_^3−^ in the HA lattice [40]. This substitution is typical in poorly crystalline, nanocrystalline, or nonstoichiometric hydroxyapatite from low-temperature, aqueous-synthesized apatites [41]), such as that seen in 3DPHA. The presence of this band reflects a less ordered HA structure where some phosphate sites are partially protonated. In addition to Ca and P, elemental analysis also showed that 3DPHA contained Na, while Mg and K were below detection limits. The Na to P molar ratio was approximately 0.17. The presence of Na further suggests Na^+^ substitution for Ca^2+^, a well-documented mechanism in non-stoichiometric and biological apatites that generates additional point defects and promotes cation vacancies [42]. Sodium substitution in apatite has also been shown to modify lattice sites related to Ca^2+^, PO_4_^2−^, and OH^−^, affecting charge compensation pathways [43]. Because Na^+^ is monovalent, its incorporation increases the overall cation deficiency and therefore enhances the requirement for HPO_4_^2−^ incorporation to maintain electroneutrality [44]. A calcium deficiency increases surface reactivity, solubility, and ion exchange capacity, factors that promote osteoconductivity and integration [45,46]. This compositional deviation also aligns 3DPHA with natural bone or naturally derived bone-graft substitutes, which are also a CDHA. This similarity is reflected in the lower-than-stoichiometric Ca/P ratios observed in commercial allografts and xenografts, as shown in Table 2. Therefore, 3DPHA is expected to be bioactive and perform well as a bone graft, which facilitates bone repair and regeneration.

Regarding heavy metal content, it was reported that heavy metal ions from biomedical implants pose significant health risks, such as allergic reactions, toxic complications, carcinogenesis, and systemic effects, including neurological and cardiovascular problems [47]. ASTM F1185 and ISO 13175 refer to USP General Chapter <232> [48], “Elemental Impurities—Limits,” for compliance standards. This USP chapter establishes stringent, health-based limits for elemental impurities potentially present in drug products, excipients, and by extension, many medical device components involved in biocompatibility assessments. For solid implantable materials, measured heavy-metal concentrations (mg/kg) are converted to Maximum Daily Dose (MDD) or Permitted Daily Exposure (PDE), considering the material mass and expected duration of human exposure. In the case of solid, long-term implantable bone grafts, it is typically assumed that the entire implant mass represents the MDD or a defined fraction thereof, particularly when gradual leaching of impurities is of concern. The daily exposure of each elemental impurity is compared against the USP <232> PDE limits for the parenteral route, which is the most relevant and strictest administration pathway for implants. Here, daily exposure was calculated by multiplying the material’s concentration by a default MDD of 10 g. This assessment yielded daily exposures of Cd, Pb, As, and Hg in 3DPHA as 0.5, 4.2, 1.1, and <0.20 μg/day, respectively. The analysis confirms full compliance of 3DPHA with USP <232> elemental impurity limits. Pb exposure is closest to its threshold of 5.0 μg/day, whereas Cd, As, and Hg show wider safety margins relative to their limits of 2.0, 15.0, and 3.0 μg/day, respectively.

Further examination revealed that while the average Pb concentration was 0.42 mg/kg (4.2 μg/day), some individual measurements reached up to 0.563 mg/kg, surpassing the specified PDE for Pb assuming 10 g/day MDD. When mean values comply but individual data points exceed regulatory limits, materials are generally regarded as non-compliant with strict surgical implant standards. Therefore, recalculation of the maximum allowable MDD, based on the highest measured Pb concentration, resulted in 8.88 g. Under this adjusted MDD, 3DPHA technically complies with USP <232> Pb limits. Given the lower concentrations of Cd, As, and Hg, they remain compliant under this threshold as well. It should be noted that USP <232> PDE values are derived from lifetime toxicological data for pharmaceuticals, assuming daily exposure over a lifetime without health effects. Bone grafts, however, are implanted once and remain long-term. If implants are treated as chronic sources of low-level exposure, impurity leaching might be averaged over a standard timeframe (often one year) or expected service life. Using single-day PDE as a compliance benchmark represents a worst-case scenario, simulating the immediate release of the entire impurity content. If the total content is below PDE limits, the implant is considered safe for chronic exposure because actual daily leaching will be substantially lower. This approach provides a conservative yet practical framework for evaluating heavy metal safety in implantable biomaterials. For comparison, the lead content in naturally derived bone grafts may be higher. This is supported by studies showing variable and sometimes elevated lead concentrations in bone tissues and hydroxyapatite derived from natural sources. Naturally sourced grafts can reflect environmental exposure and donor history, potentially resulting in higher heavy-metal content compared to synthetic or processed graft materials. An Indonesian coral *Goniopora* sp. developed as a bone graft candidate, contained As, Pb, Cd, and Hg at concentrations of 2.65, 25.23, 3.0 and 1.72 mg/kg, respectively, and the MDD for applying this coral as a bone graft was approximately 1 g [49]. A greater amount of lead contamination was also reported in both commercial allograft (mean value of 2.1 mg/kg) and xenograft (mean value of 2.0 mg/kg). A substantial proportion of specimens (38%) also contained lead concentrations in the range of 2.0 to 20.0 mg/kg [50]. This range corresponds well with the measured lead values found in the allograft and xenograft samples examined in this study, as reported in Table 2. Despite manufacturers’ assurances of purity, allografts and xenografts often contain contaminants such as donor cellular remains, cellular debris, intertrabecular fat, connective tissue, and collagen. Meanwhile, alloplastic may contain various impurities, including PVA, calcium oxide phases, calcium-deficient hydroxyapatite phases, oily substances with carbon and silicone, cellulose derivatives, alpha-tricalcium phosphate, and heavy metals [51].

The biocompatibility tests of 3DPHA demonstrate a favorable biological profile, which is important for its application as a bone-graft substitute. In vitro assessments demonstrated that 3DPHA exhibits no cytotoxic potential toward fibroblast cells while supporting the proliferation of MC3T3-E1 preosteoblasts. Although direct biological comparison with commercial allograft and xenograft materials was not feasible due to differences in physical form (granules versus disc-shaped scaffolds), the 3DPHA constructs achieved cell viability levels that exceeded the non-cytotoxicity thresholds defined by ISO 10993-5 [52]. Moreover, 3DPHA promoted osteogenic activity, as evidenced by a time-dependent increase in alkaline phosphatase (ALP) activity, a key indicator of osteoblastic differentiation. Collectively, these findings indicate that the 3DPHA scaffolds provide a favorable microenvironment for cell survival, proliferation, and differentiation, supporting their potential suitability for bone regeneration and clinical translation even in the absence of a direct commercial benchmark. Additional in vitro evaluations reveal that 3DPHA is non-irritant and non-sensitizing, minimizing risks of inflammatory or allergic reactions when implanted. Genotoxicity tests have also confirmed that the material does not induce genetic damage, ensuring its safety at the cellular genetic level. Although standard regulatory guidelines recommend a comprehensive genotoxicity including the in vitro bacterial reverse mutation (Ames) assay, the in vitro mammalian cell micronucleus (MNvit) assay, and the in vivo micronucleus (MNvivo) assay, the assessment in this study was limited to the Ames test. This was based on a tiered screening approach, prioritizing the most probable genotoxic risk associated with the fabrication method. The primary concern with 3DPHA processed via binder jetting and phase transformation is the potential for residual organic binder components to leach from the final product, especially given the low-temperature route. As the Ames test is the universally mandated and most sensitive assay for detecting gene mutations (point mutations and frameshifts), its use serves as a highly effective, high-throughput screen for these low molecular weight, DNA-reactive organic mutagens. Furthermore, the absence of any detectable organic binder residue via FTIR analysis significantly mitigates the risk of clastogenicity (chromosome breakage) and aneuploidy (chromosome loss), which are the endpoints uniquely addressed by the MNvit and MNvivo assays. Given the successful elimination of the primary source of organic genotoxicants, the likelihood of complex, large-scale chromosomal damage from minute residual inorganic elements is considered negligible compared to the risk of point mutations from potential organic components. Therefore, a negative Ames result provides preliminary evidence of genotoxic safety. Together, these findings support that 3DPHA is a biocompatible material with excellent cytocompatibility, making it promising for bone tissue engineering and clinical use in bone repair. The combination of supporting cell proliferation, promoting differentiation, and demonstrating the absence of toxicity, irritation, and sensitization underscores its suitability as a synthetic bone graft substitute in regenerative medicine.

It is important to note that the 3DPHA investigated in this study has already demonstrated favorable clinical outcomes in pilot applications for alveolar ridge preservation and bone augmentation [23,24]. These prior investigations confirmed its osteoconductivity, resorption behavior, and clinical handling performance. The present work complements those studies by providing a comprehensive toxicological and compositional validation under internationally recognized standards. Such a standardized evaluation represents a critical step in regulatory translation, ensuring that a material proven effective in pilot clinical settings also satisfies the formal chemical and biological safety criteria required for medical-device certification.

Although 3DPHA demonstrates a favorable toxicological and biological safety profile, several limitations of this study should be acknowledged. First, the in vitro models used (L929 fibroblasts and MC3T3-E1 preosteoblasts) cannot fully replicate the complexity of the in vivo environment; thus, the observed absence of cytotoxicity and promotion of osteogenic differentiation may not directly predict long-term osseointegration or immune tolerance. Second, while the in vitro irritation and sensitization assays are validated and widely accepted as alternatives to animal testing, they cannot capture the full spectrum of systemic or chronic biological responses. Third, the genotoxicity evaluation, which relied solely on the Ames test, may not completely exclude potential chromosomal or systemic genotoxic effects; complementary assays will be required for complete regulatory compliance. To address these limitations, ongoing studies by our group are investigating the long-term in vivo degradation kinetics, bone remodeling behavior, and tissue response of 3DPHA using preclinical animal models and extended clinical follow-up. Together, these efforts form a coherent translational pathway linking material innovation, toxicological assurance, and functional clinical outcomes.

## 4. Materials and Methods

### 4.1. 3D-Printed Hydroxyapatite (3DPHA) Fabrication

**Figure 4:**
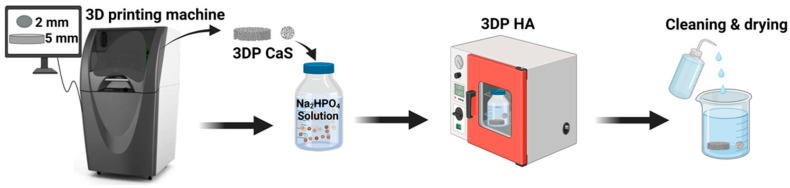
The fabrication process of 3DPHA by using binder jetting 3D printing, combined with a phase transformation process.

The 3DPHA samples used in this study were fabricated by binder jet 3D printing of calcium sulfate precursors followed by a low-temperature phase-transformation process, as described previously by our group [23,24,25,26]. In brief, calcium sulfate hemihydrate powder was printed using a water-based binder, dried, and subjected to controlled immersion in phosphate solution to achieve conversion to hydroxyapatite (Figure 4). The samples used in this work were newly prepared and characterized under the same processing conditions to ensure consistency, and all subsequent analyses were conducted independently for this study. The fundamental physical properties and mechanical properties of the 3DPHA, including SEM analysis for surface topography, porosity, pore size, and interconnectivity, have been characterized and reported in a previous publication [23,24,25,26]. In brief, the material exhibits an interconnected porous architecture with a total porosity of approximately 65.9% and an average pore size of 0.26 μm. The compressive strength (7.65 MPa) and flexural modulus and strength (1.80 GPa and 4.40 MPa, respectively) of 3DPHA were found to be comparable to those of cancellous bone, confirming its structural suitability for bone grafting applications. For comparative analysis of the Ca/P ratio and heavy metal content, a commercial bone graft material was included as a reference, including a human-derived cortical & cancellous freeze-dried bone allograft (RegenOss; granule size 0.2–1.0 mm, Neobiotech, Seoul, Republic of Korea, and Maxgraft; granule < 2.0 mm, Botiss Biomaterials, Zossen, Germany) and a bovine-derived bone xenograft (Bio-Oss; granule 1.0–2.0 mm, Geistlich Pharma, Wolhusen, Switzerland).

### 4.2. Phase and Chemical Composition

The phase composition of the 3DPHA was analyzed using X-ray diffraction (XRD, Rigaku TTRAX III, (Rigaku Corporation, Tokyo, Japan) with a Cu Kα radiation source (λ = 0.15406 nm) operating at 50 kV and 300 mA. Diffraction patterns were recorded over a 2θ range of 5–60° at a scanning speed of 3° per minute and a step size of 0.02°. Phase identification was performed using JADE software version 9.7 by comparison with reference patterns from the International Centre for Diffraction Data (ICDD), including bassanite (04-013-8392), gypsum (04-015-7420), and hydroxyapatite (HA, 01-089-4405). The relative phase content of each sample was calculated using the Rietveld refinement method in JADE. For chemical composition analysis, 3DPHA was finely ground and mixed with potassium bromide (KBr) to form KBr pellets using a compression die. Fourier Transform Infrared (FTIR) spectroscopy (Spectrum One, PerkinElmer, Shelton, CT, USA) was performed in the range of 4000–400 cm^−1^ at a resolution of 4 cm^−1^ with a TGS detector (PerkinElmer, Shelton, CT, USA).

### 4.3. Heavy Metal Quantification

3DPHA was finely ground using a mortar and pestle, and 0.025–0.075 g of the sample was dissolved in 15 mL of 1 M hydrochloric acid. The concentrations of cadmium (Cd), lead (Pb), arsenic (As), and mercury (Hg) were determined using an inductively coupled plasma mass spectrometer (ICP-MS; ICPMS-2030, Shimadzu, Kyoto, Japan). Calibration was performed using multi-element standard solutions, and all measurements were conducted in triplicate.

### 4.4. Calcium/Phosphate (Ca/P) Molar Ratio

The content of Ca, P, Na, Mg, and K was quantitatively determined by inductively coupled plasma optical emission spectrometry (ICP-OES; Optima 8000, PerkinElmer, Waltham, MA, USA). 3DPHA was finely ground using a mortar and pestle, and 0.04–0.075 g of the sample was dissolved in 15 mL of 1 M hydrochloric acid, and analyzed against standard calibration curves. Atomic % of Ca and P were obtained and calculated as Ca/P ratio.

### 4.5. Cytotoxicity

Cytotoxicity was performed following ISO 10993-5, and assessed via the MTT assay using L929 fibroblasts (1 × 10^5^ cells/mL, ATCC CCL-1, ATCC, Manassas, VA, USA) cultured in MEM (Gibco, Thermo Fisher Scientific, Waltham, MA, USA). The 3DPHA samples were extracted in 1 mL of MEM for 24 h at 37 °C. Subsequently, 100 µL of the extract was added to 200 µL of the cell suspension in a 96-well plate and incubated for 24 h. Cell viability was determined by replacing the media with 100 µL (0.5 mg/mL) MTT solution (Invitrogen, Thermo Fisher Scientific, Waltham, USA), incubating for 2 h, and dissolving the resulting formazan with 100 µL of DMSO (Sigma Aldrich, St. Louis, MO, USA). Absorbance, which correlates with the number of metabolically active cells present, was recorded at 570 nm and 600 nm using a Biochrom Asys UVM340 microplate reader (Biochrom Ltd., Cambridge, UK). The test was done in triplicate. Control groups included a negative control (Nunc™ Thermanox™ Coverslips, Thermo Fisher Scientific, Waltham, MA, USA), a positive control (polyurethane film containing 0.1% Zinc diethyldithiocarbamate (ZDEC): RM-A, Hatano Research Institute, Hatano, Kanagawa, Japan), and a blank control (wells without cells). The OD readings were subsequently used to calculate the percentage of cell viability.

Cell viability (%) = 100 × OD 570/OD 570_blank_

### 4.6. In Vitro MC3T3-E1 Cell Proliferation

The MC3T3-E1 preosteoblast cells (Subclone 4, ATCC CRL-2593, ATCC, Manassas, VA, USA) were utilized to evaluate cell compatibility and proliferation. Samples were initially pre-soaked overnight in α-MEM completed medium (Gibco^™^, Thermo Fisher Scientific, Waltham, MA, USA) before being transferred to a 48-well plate. Cells were seeded directly onto the sample surfaces at a density of 5 × 10^5^ cells/mL (100 µL per well), resulting in an initial seeding density of 5 × 10^4^ cells per sample. Cultures were maintained under standard conditions 37 °C with 5% CO_2_, 95% relative humidity). Cell proliferation on the samples was quantified at 3, 7, and 14 days using the Alamar Blue assay (Invitrogen, Thermo Fisher Scientific, USA). Prior to the assay, the culture medium was removed, and the samples were washed thoroughly three times with Dulbecco’s Phosphate-Buffered Saline (Gibco, Thermo Fisher Scientific, USA). Each sample was then incubated for 2 h with 200 µL of fresh culture medium containing 20 µL of Alamar Blue solution. The enzymatic reduction of the non-fluorescent indicator resazurin (in the Alamar Blue solution) to the highly fluorescent product resorufin by metabolically active cells was quantified by measuring absorbance. Optical density (OD) values were recorded at 570 nm and 600 nm using a microplate reader (ASYS UVM340, Biochrom Ltd., Cambridge, UK). The measured OD values provide a direct, linear indicator of cellular metabolic activity and proliferation over time.

### 4.7. ALP Activity Assay

The 3DPHA sample was placed in 24-well culture plates. MC3T3-E1 preosteoblast cells (Subclone 4, ATCC CRL-2593, ATCC, Manassas, VA, USA) were prepared at a concentration of 5 × 10^5^ cells/mL, and 100 µL of the cell suspension (equivalent to 6.5 × 10^4^ cells) was seeded onto each test sample. The plates were incubated in a CO_2_ incubator maintained at 37 °C, 5% CO_2_, and 95% relative humidity. The alkaline phosphatase (ALP) activity was measured after 3, 7, 14, and 21 days of culture. For ALP quantification, at each time point, the culture medium was removed, and the samples were rinsed three times with Dulbecco’s Phosphate-Buffered Saline (Gibco, Thermo Fisher Scientific, USA) before being transferred to new wells. Subsequently, 300 µL of sterile deionized water was added to each sample. The samples were then subjected to three freeze–thaw cycles by freezing at −80 °C for 20 min and thawing at 37 °C for 20 min per cycle. After the final cycle, 50 µL of the resulting lysate was mixed with 100 µL of p-nitrophenyl phosphate substrate solution (Alkaline Phosphatase Yellow, Sigma P7998-100 mL, St. Louis, MO, USA) and incubated at 37 °C for 15 min. The absorbance was measured at a wavelength of 405 nm using a microplate reader (ASYS UVM340, Biochrom Ltd., Cambridge, UK) to obtain OD values. The OD values, which correspond to the amount of p-nitrophenol released, were recorded to indicate the relative ALP activity of each sample. All measurements were performed in triplicate.

### 4.8. In Vitro Skin Irritation

The in vitro skin irritation test was performed using reconstructed human epidermis (RHE) models following OECD TG 439 [53]. The 3DPHA sample was extracted in 0.9% sodium chloride (NaCl) solution at a ratio of 0.2 g/mL and incubated in a 37 °C water bath shaker for 72 h. The RHE tissues (LabCytes Epi-Model 24 SIT, LabCytes Co., Ltd., Tokyo, Japan) were then incubated with the extracted sample solutions for 18 h, after which they were rinsed thoroughly with Dulbecco’s Phosphate-Buffered Saline (Gibco, Thermo Fisher Scientific, USA) to remove residual test material. Negative control (PBS) and positive control (5% sodium dodecyl sulfate, SDS solution) (Sigma-Aldrich, St. Louis, MO, USA) were employed for comparison. After incubation, 20 μL of MTS reagent (Abcam, Cambridge, UK) was added to each well, and the plates were further incubated at 37 °C for 2 h. The conversion of MTS to formazan by metabolically active cells was measured by recording the absorbance at 570 nm using a microplate reader (SpectraMax M5, Molecular Devices, San Jose, CA, USA). Percent tissue viability relative to the negative control was calculated, and the test sample was classified as irritant or non-irritant according to the OECD TG 439 criteria (viability ≤ 50% indicates irritant).

### 4.9. In Vitro Skin Sensitization

The in vitro skin sensitization test was performed following OECD TG 442E [54]. The 3DHA sample was extracted at an extraction ratio of 0.1 g/mL in Roswell Park Memorial Institute (RPMI) 1640 medium (Gibco, Thermo Fisher Scientific, Waltham, MA, USA) and incubated in a 37 °C water bath shaker for 72 h. Determination of the concentration inducing 75% cell viability (CV7) was then performed. THP-1 human monocytic leukemia cell line (ATCC TIB-202) was seeded at a density of 5 × 10^4^ cells per well in a 96-well plate. Test sample extracts were prepared at eight concentrations by 2-fold serial dilution in RPMI complete medium, ranging from 0.78–100% (*v*/*v*), resulting in final concentrations of 0.39–50% (*v*/*v*). For the positive control, 2,4-dinitrochlorobenzene (DNCB) (Sigma-Aldrich, St. Louis, MO, USA) was prepared at eight concentrations by 2-fold dilution in culture medium, ranging from 1.56–200 μg/mL, giving final concentrations of 0.71–100 μg/mL. For the negative control, lactic acid (LA) (Sigma-Aldrich, St. Louis, MO, USA) was prepared at eight concentrations ranging from 15.62–2000 μg/mL, with final concentrations of 7.81–1000 μg/mL. The culture medium (RPMI complete medium) was employed as the blank control. 80 μL of the test extract was added to each well, and the plates were incubated at 37 °C with 5% CO_2_ for 24 h. After incubation, cells were stained with propidium iodide (PI) (Sigma-Aldrich, St. Louis, MO, USA) and analyzed for cell viability (CV75) using a BD FACSLyric™ flow cytometer (BD Biosciences, San Jose, CA, USA). The experiment was conducted in duplicate.

For the determination of CD86 and CD54 expression. THP-1 human monocytic leukemia cell line (ATCC TIB-202) was seeded at a density of 1.6 × 10^5^ cells per well in a 96-well plate. The test sample concentrations were based on the CV75 value obtained from the previous assay. In cases where the test samples did not cause cytotoxicity and a CV75 value could not be determined, the maximum concentration of 50% (*v*/*v*) extract was used for testing. The extracts were added to each well at half of the initial concentrations, and the plates were incubated at 37 °C with 5% CO_2_ for 24 h. After incubation, the cells were stained with anti-CD86, anti-CD54, and anti-IgG1 antibodies, followed by propidium iodide (PI) staining to determine the percentage of viable cells. The expression levels of CD86 and CD54 were analyzed using a flow cytometer as geometric mean fluorescence intensity (MFI). The experiment was conducted in two or three independent runs (depending on the consistency of the first two runs), with duplicate samples in each run, and results were expressed as relative fluorescence intensity compared to controls. The data were used to classify the test material as sensitizing or non-sensitizing according to OECD TG 442E criteria, where a relative fluorescence intensity (RFI) value of ≥150% for CD86 and ≥200% for CD54, together with a cell viability of ≥50%, is interpreted as a positive response according to OECD TG 442E. Samples not meeting these criteria were classified as non-sensitizing.

$$RFI=\frac{\mathrm{MFI}\;\mathrm{of}\;\mathrm{chemical}-\mathrm{treated}\;\mathrm{cells}\;-\;\mathrm{MFI}\;\mathrm{of}\;\mathrm{chemical}-\mathrm{treated}\;\mathrm{isotype}\;\mathrm{control}\;\mathrm{cells}}{MFI\;of\;solvent/vehicle\;treated\;control\;cells\;-\;MFI\;of\;solvent/vehicle\;treated\;isotype\;control\;cells}\;\times \;100$$

### 4.10. Genotoxicity

The genotoxicity test was performed following OECD TG 471 [55]. The 3DPHA sample was extracted at an extraction ratio of 0.1 g/mL in Roswell Park Memorial Institute (RPMI) 1640 medium (Gibco, Thermo Fisher Scientific, Waltham, MA, USA) and incubated in a 37 °C water bath shaker for 72 h. The bacterial reverse mutation (Ames) assay was employed to evaluate genotoxicity. *Salmonella typhimurium* strains TA1535 (ATCC 11325), TA1538 (ATCC 14028), TA98 (ATCC 12026), TA100 (ATCC 12027), and TA102 (ATCC 29621) were obtained from American Type Culture Collection (ATCC, USA) and inoculated into nutrient broth (NB) medium (Thermo Fisher Scientific company, Waltham, MA, USA). The cultures were incubated at 37 ± 2 °C for 16–24 h to prepare actively growing bacterial cultures for the assay. The bacterial cultures were then diluted with Dulbecco’s Phosphate-Buffered Saline (Gibco, Thermo Fisher Scientific, USA) to obtain a final concentration of approximately 10^8^ CFU/mL, which was used for the subsequent assays. Two milliliters of nutrient broth were pipetted into a test tube containing 100 μL of an Ames strain and 200 μL of the extracted test sample. The mixture was vortexed thoroughly and poured onto a GM plate (Vogel-Bonner Salts Medium) (Fisher Scientific, Waltham, MA, USA). The test was performed in triplicate. The plates were incubated at 37 ± 2 °C for 48–72 h, after which the number of colonies formed on each plate was counted. The results were expressed as the number of revertant colonies counted on each plate and used to calculate the mutagenic index (MI) according to the formula:

$$MI=\frac{\mathrm{Number}\;\mathrm{of}\;\mathrm{colonies}\;\mathrm{with}\;\mathrm{test}\;\mathrm{extract}}{\mathrm{Number}\;\mathrm{of}\;\mathrm{colonies}\;\mathrm{in}\;\mathrm{negative}\;\mathrm{control}}$$

A mutagenic index (MI) ≥ 2 indicates that the test substance is mutagenic, whereas an MI < 2 indicates no mutagenic activity was observed. Phosphate-buffered saline (PBS) was used as the negative control, while sodium azide (Sigma-Aldrich, St. Louis, MO, USA) was used as the positive control for *Salmonella typhimurium* TA1535 and TA100, 4-nitro-o-phenylenediamine (Sigma-Aldrich, St. Louis, MO, USA) for TA1538, 2-nitrofluorene (Sigma-Aldrich, St. Louis, MO, USA) for TA98, and mitomycin C (Sigma-Aldrich, St. Louis, MO, USA) for TA102.

## 5. Conclusions

This study provides a comprehensive toxicological and compositional safety evaluation of 3DPHA fabricated by our group through binder-jet 3D printing of calcium sulfate precursors followed by low-temperature phase transformation. While the fabrication route and preliminary clinical performance of 3DPHA have been reported previously, the material’s chemical purity, elemental safety, and in vitro biocompatibility had not been systematically examined. The present results confirm that 3DPHA is chemically stable, free of hazardous impurities, and biologically safe, meeting the relevant ISO 10993 and OECD test criteria. Combined with our earlier preclinical and pilot clinical findings, this toxicological validation provides the missing regulatory and scientific foundation supporting the material’s safety for broader clinical translation. Future studies will extend this work to examine long-term in vivo degradation, bone-repair efficacy, and tissue-integration behavior, completing the translational assessment of 3DPHA as a bone-graft substitute.

## Figures and Tables

**Table 1:** Phase composition percentage of the as-printed sample and 3DPHA (Mean ± SD).

Samples	Phase Composition (%)		
	Bassanite	Gypsum	Hydroxyapatite
As-printed	86.6 ± 8.5	13.4 ± 8.5	-
3DPHA	-	-	100

**Table 2:** Heavy metal content and Ca/P molar ratio of 3DPHA and commercial bone grafts (Mean ± SD).

Samples	Heavy Metal (mg/kg)				Ca/P Molar Ratio
	Cd	Pb	As	Hg	
3DPHA	0.05 ± 0.02	0.42 ± 0.17	0.11 ± 0.04	<0.020	1.38 ± 0.03
Maxgraft	<0.020	1.86	<0.020	<0.020	1.53
RegenOss	<0.020	2.38	0.03	<0.020	1.52
Bio-Oss	<0.020	0.85	0.24	<0.020	1.50

**Table 3:** Cell viability percentage of the 3DPHA by using MTT assay (Mean ± SD).

Samples	Cell Viability (%)
Blank	100.0 ± 0.00
Positive	0.0 ± 0.00
Negative	100.1 ± 0.87
3DPHA	82.4 ± 1.91

**Table 4:** Tissue viability percentage of the 3DPHA by using the MTS assay in the irritation test (Mean ± SD).

Samples	% Tissue Viability
Negative	100.0 ± 6.0
Positive	15.7 ± 4.1
3DPHA	99.0 ± 1.9

**Table 5:** Dose finding assay for CV75 (Mean ± SD).

LA (μg/mL)		DNCB (μg/mL)		3DPHA (*v*/*v*)	
Concentration	Cell Viability (%)	Concentration	Cell Viability (%)	Concentration	Cell Viability (%)
0	96.7 ± 0.9	0	96.7 ± 0.9	0	96.7 ± 0.9
7.8	95.8 ± 0.5	0.78	93.5 ± 1.3	0.39	93.7 ± 0.3
15.63	94.0 ± 0.4	1.56	89.6 ± 0.8	0.78	94.0 ± 0.1
31.25	96.2 ± 0.6	3.13	84.9 ± 1.9	1.56	93.2 ± 0.1
62.5	95.8 ± 1.3	6.25	31.7 ± 0.0	3.13	92.7 ± 0.4
125	96.0 ± 0.1	12.5	31.6 ± 0.4	6.25	89.8 ± 2.0
250	96.1 ± 0.4	25	28.8 ± 0.8	12.5	94.2 ± 0.7
500	95.1 ± 1.2	50	26.9 ± 0.4	25	91.9 ± 0.1
1000	95.3 ± 2.1	100	28.8 ± 1.8	50	91.6 ± 2.5

**Table 6:** In Vitro Skin Sensitization of 3DPHA (Mean ± SD).

Samples	Concentration	CD86		CD54	
		Cell Viability (%)	RFI	Cell Viability (%)	RFI
Blank	0	96.0 ± 0.8	100.0 ± 0.0	96.7 ± 0.3	100.0 ± 0.0
Negative	1000 μg/mL	96.1 ± 0.4	89.9 ± 26.1	96.1 ± 1.6	113.3 ± 45.0
Positive	3.55 μg/mL (CV75)	91.7 ± 0.9	202.5 ± 16.5	90.9 ± 0.9	526.4 ± 89.2
3DPHA	50 *v*/*v*	94.4 ± 2.5	99.3 ± 16.1	95.5 ± 1.6	141.8 ± 71.3

**Table 7:** In Vitro Mutagenic Index (MI) of 3DPHA.

MI	Ames Strains				
	TA1535	TA1538	TA98	TA100	TA102
Negative	1.00	1.00	1.00	1.00	1.00
Positive	3.97	5.88	3.00	5.90	3.10
3DPHA	1.14	0.75	1.00	0.59	0.84

## Data Availability

The original contributions presented in this study are included in the article. Further inquiries can be directed to the corresponding author.

## Abbreviations

HA	Hydroxyapatite
CDHA	Calcium-Deficient Hydroxyapatite
3DPHA	3D-Printed Hydroxyapatite
PVA	Polyvinyl Alcohol
ACP	Amorphous Calcium Phosphate
SLA	Stereolithography
FDM	Fused Deposition Modelling
XRD	X-ray Diffraction
FTIR	Fourier Transform Infrared Spectroscopy
SEM	Scanning Electron Microscopy
EDS	Energy Dispersive Spectroscopy
Ca/P	Calcium/Phosphate Ratio
ICP-OES	Inductively Coupled Plasma Optical Emission Spectrometry
ICP-MS	Inductively Coupled Plasma Mass Spectrometry
MDD	Maximum Daily Dose
PDE	Permitted Daily Exposure
MEM	Minimum Essential Medium
α-MEM	Minimum Essential Medium Eagle—Alpha Modification
DMEM	Dulbecco’s Modified Eagle Medium
MTT	3-(4,5-Dimethylthiazol-2-yl)-2,5-Diphenyltetrazolium Bromide
MTS	3-(4,5-Dimethylthiazol-2-yl)-5-(3-Carboxymethoxyphenyl)-2-(4-Sulfophenyl)-2H-Tetrazolium
ALP	Alkaline Phosphatase
BM-MSCs	Bone Marrow-derived Mesenchymal Stem Cells
RHE	Reconstructed Human Epidermis
DMSO	Dimethyl Sulfoxide
PBS	Phosphate-Buffered Saline
OD	Optical Density
OECD	Organisation for Economic Co-operation and Development
RPMI	Roswell Park Memorial Institute
DNCB	2,4-Dinitrochlorobenzene
LA	Lactic Acid
SDS	Sodium Dodecyl Sulfate
PI	Propidium Iodide
MI	Mutagenic Index
